# Oncogenic Osteomalacia as the Initial Presentation of Pleural Epithelioid Hemangioendothelioma: A Case Report

**DOI:** 10.7759/cureus.75495

**Published:** 2024-12-10

**Authors:** Najla S Ewain, Sameerah Alshehri, Majed M Aladwani

**Affiliations:** 1 Department of Medicine, Division of Endocrinology and Metabolism, King Abdulaziz Medical City, Riyadh, SAU; 2 Department of Medicine, King Abdullah International Medical Research Center, Riyadh, SAU; 3 Department of Medicine, College of Medicine, King Saud Bin Abdulaziz University for Health Sciences, Riyadh, SAU

**Keywords:** epithelioid hemangioendothelioma, fgf-23, hypophosphatemia, oncogenic osteomalacia, paraneoplastic syndrome

## Abstract

Epithelioid hemangioendothelioma (EHE) is a rare form of vascular neoplasm that can manifest with various symptoms or be discovered incidentally in asymptomatic patients. In this report, we describe a case of a 56-year-old male who presented with progressive lower limb weakness over four years. The evaluation revealed severe hypophosphatemia, an inappropriately normal fibroblast growth factor 23 C-terminal (cFGF-23) level, and a 30 x 20 mm hypermetabolic right pleural mass, which was subsequently proven to be EHE. Following tumor resection, the patient experienced complete recovery, with normalization of his serum phosphate levels and resolution of his presenting symptoms. This case highlights the association between EHE and oncogenic osteomalacia, a rare paraneoplastic syndrome.

## Introduction

Oncogenic osteomalacia, also known as tumor-induced osteomalacia, is a rare paraneoplastic syndrome characterized by hypophosphatemia and impaired bone mineralization secondary to renal phosphate wasting. This condition closely resembles the clinical and biochemical profile of genetic forms of hypophosphatemic rickets [1]. Elevated levels of fibroblast growth factor 23 (FGF-23) have been demonstrated in tumors from patients with oncogenic osteomalacia and in patients with X-linked hypophosphatemia, leading to increased urinary phosphate loss [2].

Epithelioid hemangioendothelioma (EHE) is a rare vascular neoplasm composed of epithelioid or histocytoid cells with endothelial characteristics. With an incidence of approximately one per million, EHE most commonly arises in the liver, followed by the lungs and bones, and is rarely reported as a localized pleural mass [3].

In this report, we describe a case of oncogenic osteomalacia linked to a pleural tumor with the histopathological finding of EHE, highlighting an unusual manifestation of this rare disease. To our knowledge, the association between EHE and oncogenic osteomalacia has only been reported once in the literature to date [4].

## Case presentation

A 56-year-old male with a medical history of hypertension and a remote history of cervical spine surgery in 2016 was referred from a smaller hospital to our tertiary hospital in 2021 for decompression surgery of L4-L5 vertebral stenosis. The patient presented to the clinic in a wheelchair, reporting a four-year history of progressive muscle weakness that began proximally and subsequently became generalized, ultimately impairing his ability to walk. He denied any sensory loss, numbness, history of trauma, urinary or bowel incontinence, or urinary retention. There was no history of fever, unintentional weight loss, or corticosteroid use.

Upon examination, the patient had a Glasgow Coma Scale score of 15/15, and all vital signs were within normal limits. He was sitting in a wheelchair. Examination of the cardiovascular, respiratory, and gastrointestinal systems was normal. Neurological evaluation revealed normal cranial nerve function and intact bilateral upper and lower limb sensation (C5-T1). However, muscle power was significantly reduced, ranging from 3 to 4 out of 5 in the upper limbs and 2 to 4 out of 5 in the lower limbs bilaterally.

The patient was admitted for orthopedic evaluation, as his symptoms were not explained by the L4-L5 stenosis.

Laboratory findings showed normal complete blood count, renal function, liver enzymes, and thyroid function. He had a normal 25-hydroxy vitamin D level of 73.4 nmol/L, a normal adjusted calcium level of 2.12 mmol/L, a normal magnesium level of 0.76 mmol/L, normal potassium and sodium levels, elevated alkaline phosphatase of 760 U/L, and a low phosphorus level of 0.28 mmol/L. Parathyroid hormone level was mildly elevated at 10.860 pmol/L, and 1,25-dihydroxy vitamin D level was low at 20.8 pmol/L. Further investigations revealed negative tumor markers (namely, carcinoembryonic antigen (CEA), prostate-specific antigen (PSA), alpha-fetoprotein (AFP), carbohydrate antigen 19-9 (CA 19-9), cancer antigen 125 (CA 125), and cancer antigen 15-3 (CA 15-3)) and negative biochemical markers for multiple myeloma (Table 1).

**Table 1:** Laboratory values upon initial presentation

Test	Result	Reference range	Unit
Hemoglobin	141	135-180	gm/L
Red blood cells	4.53	4.5-6.1	x10^12/L
Mean corpuscular volume (MCV)	91.3	76-96	fL
White blood cells	4.41	4-11	x10^9/L
Platelet	193	150-400	x10^9/L
Creatinine	64	64-110	umol/L
Estimated glomerular filtration rate	118	60	mL/min/1.73 m^2^
Alanine transferase	14	5-55	U/L
Aspartate aminotransferase	18	5-34	U/L
Gamma-glutamyl transferase	17.4	12-64	U/L
Total bilirubin	10.6	20.5	umol/L
Thyroid-stimulating hormone	3.44	0.35-4.94	mIU/L
Free T4	9.84	9.00-19.00	pmol/L
25-hydroxy vitamin D	73.4	50-125	nmol/L
Adjusted calcium	2.12	2.1-2.55	mmol/L
Magnesium	0.76	0.66-1.07	mmol/L
Potassium	4.3	3.5-5.1	mmol/L
Sodium	139	136-145	mmol/L
Phosphorus	0.28	0.74-1.52	mmol/L
Alkaline phosphatase	760	40-150	U/L
Parathyroid hormone	10.860	1.950-7.240	pmol/L
1,25-dihydroxy vitamin D	20.8	62.6-228	pmol/L
Carcinoembryonic antigen (CEA)	2.1	5	ng/mL
Prostate-specific antigen (PSA)	0.490	4.000	ug/L
Alpha-fetoprotein (AFP)	2.6	8.8	ng/mL
Carbohydrate antigen 19-9 (CA 19-9)	2	37	U/mL
Cancer antigen 125 (CA 125)	15	35	U/ml
Cancer antigen 15-3 (CA 15-3)	9.3	31.3	U/mL
Serum protein electrophoresis - M spike	0 g/L, 0%	0 g/L, 0%	-
Bence Jones protein	Absent	Absent	-

The patient required daily phosphate replacements, both orally and occasionally intravenously, due to a critically low phosphate level.

Radiological imaging showed evidence of previous instrumentation at the C4-C5 level, diffuse trabecular thickening in the cervical and thoracolumbar spine on a background of osteoporotic bone, and a variable degree of vertebral body height loss, mainly at the lumbar spine. Diffuse increased trabecular thickening was also noted at the upper and lower extremities, with evidence of ongoing demineralization. Old fractures were noted at the proximal tibia bilaterally, with generalized prominent skeletal activity and multiple other fractures, including rib and sacral fractures, on a background of reduced bone density with focal lucencies.

The spinal cord and craniocervical junction appeared unremarkable, with no signs of spinal cord compression or significant spinal canal stenosis.

**Figure 1:**
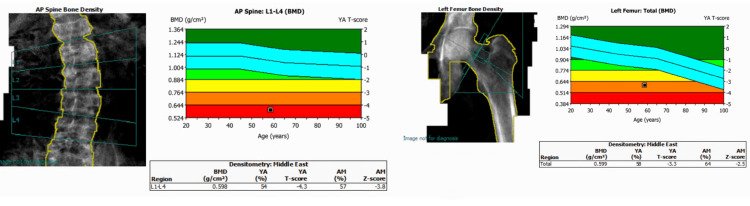
Baseline bone densitometry scan. BMD, bone mineral density

A bone mineral density (BMD) scan reported a lumbar spine BMD of 0.598 g/cm², which represents a T-score of -4.3; a left proximal femur BMD of 0.599 g/cm², which represents a T-score of -3.3; and a left femoral neck BMD of 0.626 g/cm², which represents a T-score of -3.1 (Figure 1).

Differential diagnoses initially considered included a paraneoplastic syndrome, Paget disease of bone, and osteoporosis. The alkaline phosphatase level was notably elevated at 760 U/L (reference range: 40-150). Further investigations to rule out secondary causes of osteoporosis revealed an average testosterone level and a normal response to the dexamethasone suppression test, in addition to the previously mentioned results. The patient received one dose of 4 mg IV zoledronic acid.

**Figure 2:**
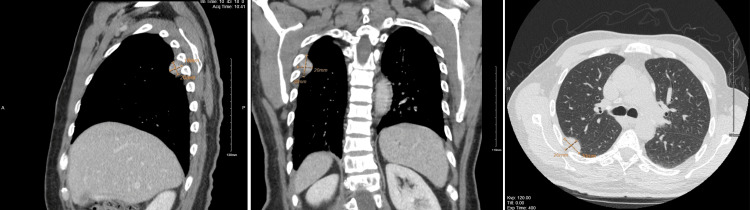
Chest CT findings of the right pleural mass, measuring 30 x 20 mm in maximum dimensions. They are shown in sagittal, coronal, and axial views. CT, computed tomography

As part of the malignancy workup, a chest CT scan was done and revealed a well-defined, oval, right upper pleural-based soft tissue lesion measuring approximately 30 x 20 mm (Figure 2). There was no adjacent bone destruction, lung invasion, or suspicious pulmonary nodules or consolidation.

A computed tomography (CT)-guided biopsy of the mass was obtained, the pathology report revealed a low-grade spindle cell neoplasm, positive for ERG antibody, suggestive of EHE.

**Figure 3:**
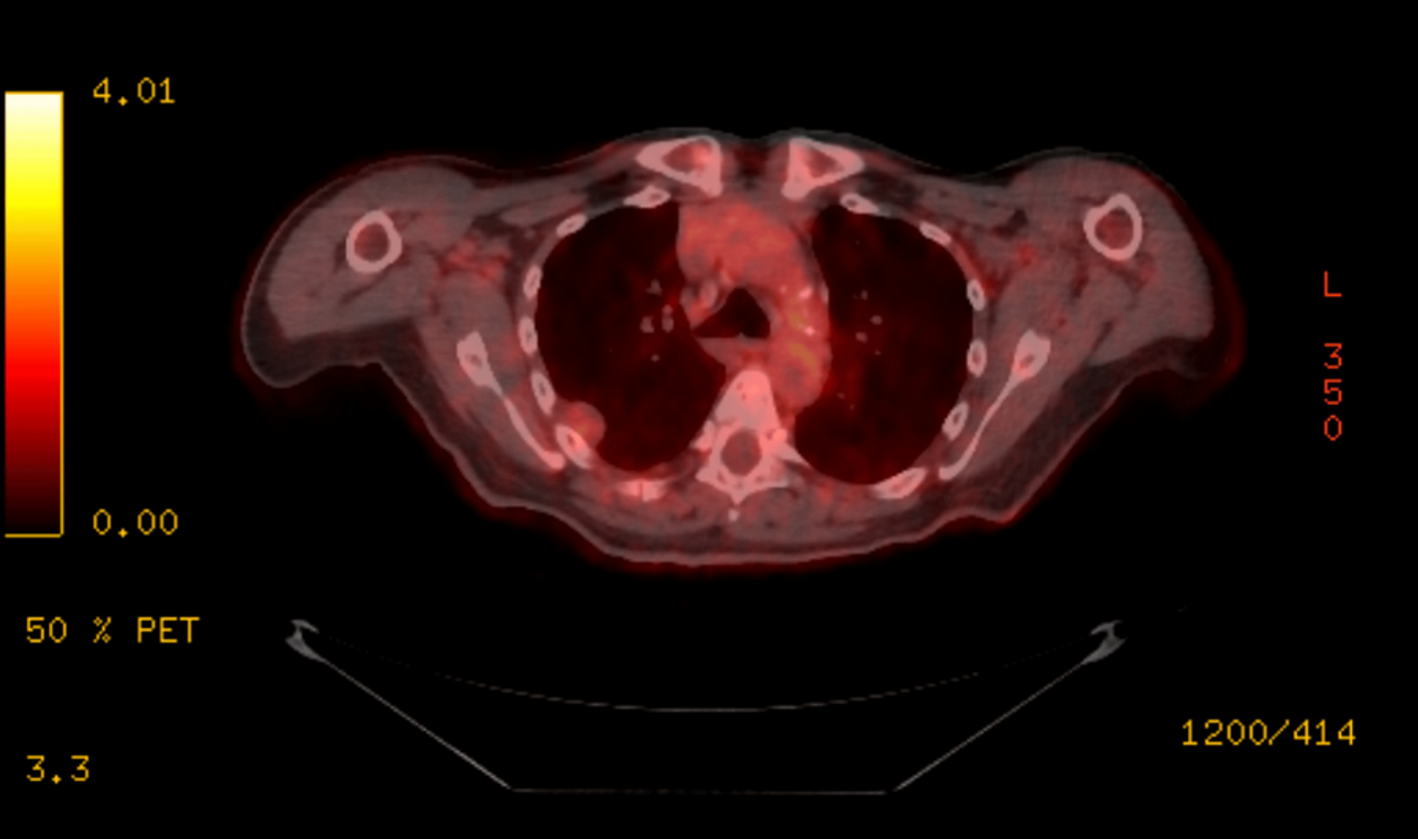
An 18-FDG PET/CT finding of a hypermetabolic, mildly active right upper pleural-based nodule, with the highest SUV of 3.8. FDG, fluorodeoxyglucose; SUV, standardized uptake value; PET/CT, positron emission tomography/computed tomography

Following the pathological findings, an 18-fluorodeoxyglucose (18-FDG) positron emission tomography (PET)/CT scan was performed to assess for metastasis. It showed a hypermetabolic, mildly active right upper pleural-based nodule measuring 17 x 21 mm, with the highest standardized uptake value (SUV) of 3.8 (Figure 3), consistent with the biopsy-proven EHE. There was no evidence of hypermetabolic pulmonary or extrathoracic disease.

Given the significant hypophosphatemia in the presence of a pleural mass, the suspicion of oncogenic osteomalacia was raised, prompting a request for the FGF-23 level. The FGF-23 C-terminal (cFGF-23) level came back as 79 kRU/L (reference range: 26-110). This result was interpreted as inappropriately normal, given the severity of hypophosphatemia. A 24-hour urine phosphate level was also requested; however, it was deemed inaccurate due to the patient being on phosphate replacement and improper collection.

**Figure 4:**
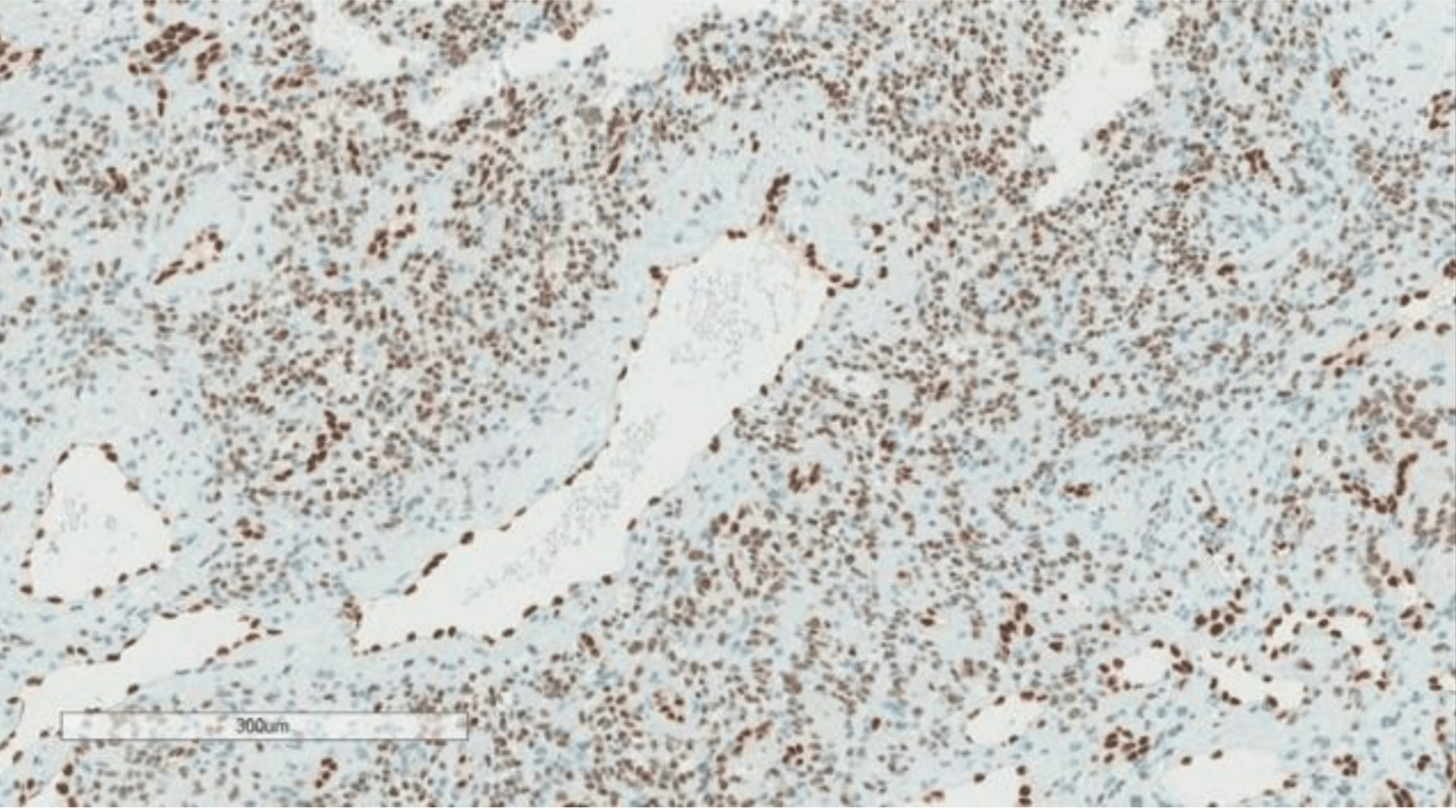
A low-power immunohistochemical stain for the ERG antibody is positive in the spindle cells and endothelial cells of the large vessels. ERG, ETS family transcription factor ERG

The patient underwent right video-assisted thoracoscopic surgery (VATS) with wide local excision of the right upper pleural mass. The pathological findings confirmed the diagnosis of EHE, and the spindle cells were positive for the ERG antibody, a specific nuclear marker for this lesion (Figure 4). The pathological details of this pleural EHE have been reported separately by our colleagues from the Pathology Department [5]. The genomic finding of BAP1 rearrangement in intron 6 was detected and reported as a variant of unknown significance at the time of testing.

The tumor board discussed the case and decided against cytotoxic therapy initially, as the tumor was considered resectable.

After tumor resection, serum phosphate levels improved to above 1 mmol/L without supplements (reference range: 0.74-1.52), the patient’s clinical symptoms resolved, and he was able to walk unaided within a few weeks post-surgery. Approximately seven weeks later, the cFGF-23 level decreased from 79 to 22 kRU/L (reference range: 26-110).

Currently, the patient is being followed with chest, abdomen, and pelvis CT scans every 6 to 12 months, with no evidence of disease recurrence or metastasis noted during active surveillance.

**Figure 5:**
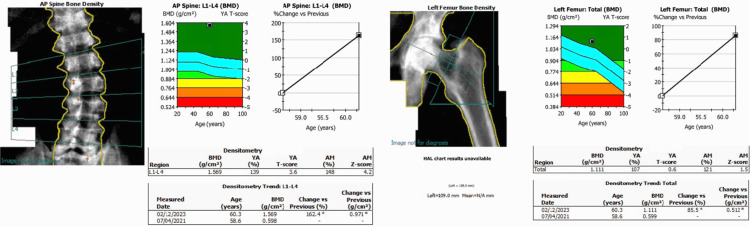
Bone densitometry scan performed two years post-tumor resection, showing interval changes compared to the previous scan. BMD, bone mineral density

His BMD was re-evaluated two years after the surgery, showing significant improvement, with a 162.4% increase in lumbar spine BMD and an 85.5% increase in proximal femur BMD compared to the pre-surgery scan (Figure 5).

## Discussion

This case report involved a patient with predominantly lower limb weakness. Further, the workup revealed severe hypophosphatemia, a 30 x 20 mm hypermetabolic right pleural mass, and an inappropriately normal cFGF-23 level. These findings are consistent with a paraneoplastic syndrome known as oncogenic osteomalacia.

Oncogenic osteomalacia, also known as tumor-induced osteomalacia, is a rare endocrinological paraneoplastic syndrome characterized by hypophosphatemia and abnormal bone mineralization, secondary to renal phosphate losses. It closely resembles the biochemical, imaging, and clinical findings in those with genetic forms of hypophosphatemic rickets [1].

High levels of FGF-23 have been observed in tumors from patients with oncogenic osteomalacia and in X-linked hypophosphatemia. FGF-23 is a polypeptide that reduces phosphate reabsorption and decreases the kidney's production of 1,25-dihydroxy vitamin D, resulting in urinary phosphate loss and hypophosphatemia [1,2].

The biochemical hallmark of oncogenic osteomalacia is hypophosphatemia in the presence of phosphaturia and low or abnormally normal levels of 1,25-dihydroxy vitamin D. Other biochemical findings include normal serum calcium and elevated alkaline phosphatase levels. Radiologically, it manifests as severe osteomalacia [1,6]; nearly all of these findings were present in the case reported here.

Once oncogenic osteomalacia is suspected, locating the culprit tumor can be challenging due to its typically small size and obscure location. In cases of high suspicion without tumor identification via conventional imaging, alternative scanning techniques, including whole-body magnetic resonance imaging (MRI), 18-FDG-PET/CT, or indium 111-pentetreotide scintigraphy, should be considered [7]. Fortunately, in our case, the tumor was readily localized on a contrast-enhanced chest CT scan.

Common histological entities associated with this paraneoplastic syndrome include hemangiopericytoma, non-ossifying fibroma, giant cell tumor, and hemangioma [8]. However, the EHE identified in this case has not previously been well-recognized as being associated with oncogenic osteomalacia.

The definitive treatment of oncogenic osteomalacia is resection of the culprit tumor, which typically leads to prompt biochemical and bone recovery within 6 to 12 weeks [1]. Medical therapy is indicated if the tumor cannot be identified or completely resected. Burosumab, an anti-FGF-23 monoclonal antibody, is the preferred therapy.

In the case presented here, the tumor was resected and histopathologically confirmed to be EHE, with the right side of the pleura being the primary site and no evidence of metastasis. The patient exhibited dramatic improvement in both clinical and biochemical findings post-resection, confirming the association between his oncogenic osteomalacia presentation and the diagnosis of pleural EHE in his case. To date, the patient has not shown evidence of tumor recurrence or metastasis post-resection. To the best of our knowledge, the association between oncogenic osteomalacia and EHE has been reported only once in the literature; similar to our case, it was reported to arise from the mediastinum [6].

## Conclusions

Oncogenic osteomalacia is a rare paraneoplastic syndrome, not well recognized as being associated with EHE tumors. Clinicians should remain vigilant for signs of oncogenic osteomalacia in patients presenting with unexplained muscle weakness and BMD loss, especially in the context of a known malignancy. A high index of clinical suspicion is critical for diagnosing and managing such cases.
